# Rodent–Human Interface: Behavioral Risk Factors and Leptospirosis in a Province in the Central Region of Thailand

**DOI:** 10.3390/vetsci9020085

**Published:** 2022-02-17

**Authors:** Kanokwan Suwannarong, Ngamphol Soonthornworasiri, Pannamas Maneekan, Surapon Yimsamran, Karnsunaphat Balthip, Santi Maneewatchararangsri, Watcharee Saisongkorh, Chutarat Saengkul, Suntaree Sangmukdanun, Nittaya Phunta, Pratap Singhasivanon

**Affiliations:** 1 Department of Tropical Hygiene, Faculty of Tropical Medicine, Mahidol University, Ratchathewi, Bangkok 10400, Thailand; kanokwan27@yahoo.com (K.S.); pannamas.man@mahidol.ac.th (P.M.); addy.yim@gmail.com (S.Y.); suntaree.san@mahidol.edu (S.S.); pratap.sin@mahidol.ac.th (P.S.); 2 SUPA71 Co., Ltd., Bangkok 10230, Thailand; 3 Faculty of Nursing, Prince of Songkla University, Hat Yai 90110, Thailand; quantar.b@psu.ac.th; 4 Department of Molecular Tropical Medicine and Genetics, Faculty of Tropical Medicine, Mahidol University, Bangkok 10400, Thailand; santi.man@mahidol.ac.th; 5 Department of Medical Sciences, Ministry of Public Health, Muang District, Nonthaburi 11000, Thailand; watcharee.s@dmsc.mail.go.th; 6 Faculty of Public Health, Nakhon Sawan Campus, Mahidol University, Nakhon Sawan 60130, Thailand; chutarat.san@mahidol.ac.th; 7 Ban Dan Health Promotion Hospital (under Ministry of Public Health Thailand), Ban Phot Pisai District, Nakhon Sawan 60180, Thailand; n.phunta@gmail.com

**Keywords:** contact, interface, Nakhon Sawan, rodents, Thailand, wildlife

## Abstract

This sequential explanatory mixed-method study consisted of analytical, cross-sectional, and qualitative studies. The research was conducted in the Khao Nor and Khao Kaew areas of the Banphot Pisai districts of Nakhon Sawan Province in 2019. Here, we examined the rodent contact characteristics of villagers in these areas and determined the potential characteristics/risk factors associated with rodents using a semi-structured questionnaire, key informant interview (KII), and focus group discussion (FGD). Results of the quantitative study (N1 = 372) characterized participants that contacted rodents per gender, age, occupation, knowledge, attitude, and practice (KAP), including their cultural contexts, and beliefs. Ninety participants (24.2%) reported contact with rodents, and the reasons for their direct physical rodent contact were hunting (35, 9.4%), killing (41, 11.0%), preparing rodents as food (33, 8.9%), consuming cooked meats (12, 3.2%), feeding food (4, 1.1%), cleaning feces (17, 4.6%), and cleaning carcasses (33, 8.9%). Moreover, logistic regression results showed that males encountering rodents were statistically significant (Adjusted OR = 3.137, 95% CI 1.914–5.139, *p* < 0.001). Low monthly household income (<THB 15,000 or <USD 450) was also negatively statistically significant with encountering rodents (Adjusted OR = 0.57, 95% CI 0.33–0.99, *p* = 0.04). Additionally, the villagers had a low level of knowledge toward zoonotic diseases and inappropriate attitudes and practices toward contacting rodents and zoonotic diseases. Thirty-five qualitative study participants (N2) participated in the KIIs and FGDs. Various rodent contact activities were also reported among the qualitative research participants, such as hunting, consumption, and selling them to their friends and neighbors. However, these rodents also destroyed their belongings, crops, and plants. Some participants also reported that rodents accounted for leptospirosis transmission. As a result, communication intervention should be planned to provide appropriate knowledge and attitude to the villagers, especially among those who have close contact with rodents in the understudied area.

## 1. Introduction

Emerging Infectious Diseases (EIDs) are a burden to the worldwide health condition [1], and they are caused by at least 12% of all human pathogens [2]. Zoonotic pathogens account for about three-fourths of human EIDs [3], of which the majority of these (71.8%) originate from wildlife and increase significantly over time [4].

Hence, important zoonotic diseases (ZDs) from wildlife include avian influenza [5], Ebola [6], food poisoning [7], Hantavirus [8], leptospirosis [9], lyssavirus [10], Nipah virus [11], rabies [12], severe acute respiratory syndrome (SARS) [13], parasitic diseases [14,15], plague [16], and toxoplasmosis [17]. Some ZDs mentioned above are from wildlife reservoirs, such as rodents, which present important health issues, thereby affecting all from place to place worldwide [18]. Wildlife can also be used for several purposes, such as hunting [19], bushmeat consumption [20], traditional medicine [21], natural insect control [22], sale, or trade [23]. Additionally, wildlife guano (feces) is used as fertilizer [24]. Moreover, human behavior, knowledge, belief, cultural context, and sociodemographic characteristics are potential factors that are linked with wildlife contact activities [25].

Leptospirosis is among the ZDs that have accounted for a high number of public health issues for the past two decades worldwide, especially in resource-poor and developing countries, including Thailand [9]. Leptospirosis can be acquired through contact with domestic animals and wildlife, e.g., dogs [26], cats [27], cattle [28], and rodents [29], which are considered reservoirs for leptospirosis infection in humans.

Thailand has several urban and rural settings with a high density of wildlife nearby and around their communities (urban, peri-urban, and rural settings). Villagers would therefore have a great chance to contact wildlife, which exposes them to zoonotic diseases. Thailand also has several places where wildlife resides near the communities, and people in these communities frequently have contact with wildlife during several activities. However, the factors and characteristics of contact with wildlife remain doubtful, and only a few have been studied. In addition, the wildlife-contacting prevalence rate of the Thai people is unknown, especially in the central region of Thailand. Therefore, this study was initiated in the Banphot Pisai district, Nakhon Sawan province, a central Thai region with unique characteristics, including various domestic and wildlife species, such as rodents, bats, and non-human primates in the same areas. According to the scoping visit observations, people in this district also have close contact with these animals. The study team also learned that the areas had been affected by flooding in 2008, and health statistics showed a number of fever cases of unknown origin annually.

Additionally, the villagers have agriculture-related occupations that might lead to leptospirosis infection. Therefore, this study used leptospirosis as a “proxy indicator” to determine the animal exposure activities of the villagers by obtaining seroprevalence rates. Hence, this study determined the potential risk factors associated with coming into contact with various rodent types. This study also examined the factors related to rodent-contacting behaviors among villagers who lived in the study areas at least twelve months before data collection in 2019.

## 2. Materials and Methods

### 2.1. Study Design

A sequential explanatory mixed-method study design was conducted, which consisted of an analytical cross-sectional study to determine the seroprevalence of leptospirosis among healthy subjects and a qualitative study. Results of the qualitative study were then used to support findings from the quantitative study and provide recommendations for further studies and interventions.

### 2.2. Study Sites

The Banphot Pisai district is one of fifteen districts in Nakhon Sawan province that was selected as the study area. It is located in the neighboring Kamphaeng Phet and Phichit provinces. This district was chosen because it has rows of mountains, namely, Khao Nor and Khao Kaew. The mountains also have forests and caves that several types of wildlife live in and around, especially rodents, bats [30], non-human primates, and villagers, thereby facilitating direct and indirect physical contact activities with wildlife animals per observation by the study researcher during several scoping visits. People in the areas worked in agriculture-related occupations, such as rice field farmers, who could become prone to contact with rodents’ carriers for leptospirosis. The study results were also used to compare with previous studies in the Khon Kaen province, a northeastern region of Thailand [31], and Lao PDR [32] on rodent contact and consumption behavior characteristics, and whether their knowledge, attitudes, perceptions, and practices were different from those of the villagers who live in the central region of Thailand.

**Figure 1:**
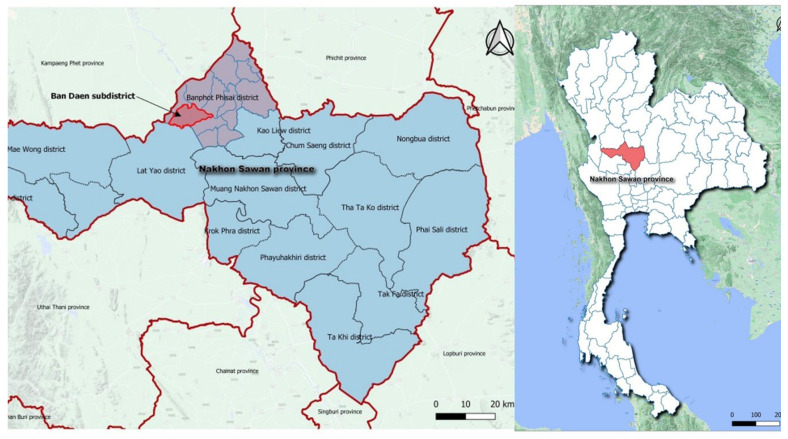
Maps of the Banphot Pisai District, Nakhon Sawan province.

Maps of the province, district, and subdistrict, including photos of the environmental characteristics of the district, are shown in Figure 1.

### 2.3. Study Participant Criteria

The participant criteria comprised individuals aged between 20 and 65 years who had lived in the areas for at least twelve months before the study implementation and who were willing to participate in this study.

### 2.4. Sample Size Estimation

#### 2.4.1. Analytic Cross-Sectional Survey and Blood Specimen Collection

The following standard formula was used for calculating the minimum sample size:

$$n=\frac{z^{2}\;p\left(1-p\right)}{d^{2}}$$

Here, *p* = proportion of the indicator of interest, based on the previous study by Thipmontree et al. in northeastern Thailand [33]. This study was used to calculate the proportion since there are few numbers of studies on leptospirosis in the central region of Thailand. The study found that 40% of acute undifferentiated fevers were diagnosed as leptospirosis infections. Therefore, *p* was 0.40 for this calculation. *z* = 1.96 (95% confidence interval), and *d* (margin of error) *=* 5%. The sample size for this quantitative survey was 369 as a minimum number.

Our study populations were selected randomly using the most updated household registry record database from a health-promoting hospital (HPH) in the study area. Since a possible decline in participation was expected, a 20% pre-random selection was made to fulfill the required sample size numbers. Finally, 443 participants were pre-selected from the database and contacted for the interviews for this quantitative survey, in which a total of 372 people agreed to participate in the quantitative study.

#### 2.4.2. Qualitative Study

For the qualitative study, key informant interviews (KIIs) and focus group discussions (FGDs) were conducted. The participants were engaged in purposeful discussions following selections as per suggestion from the village chiefs or local health authorities of those who were highly experienced with having close contact with rodents in the study areas, including those who reported close contact behaviors with rodents in the analytical cross-sectional survey.

### 2.5. Study Tools and Data Collection Procedures

**Figure 2:**
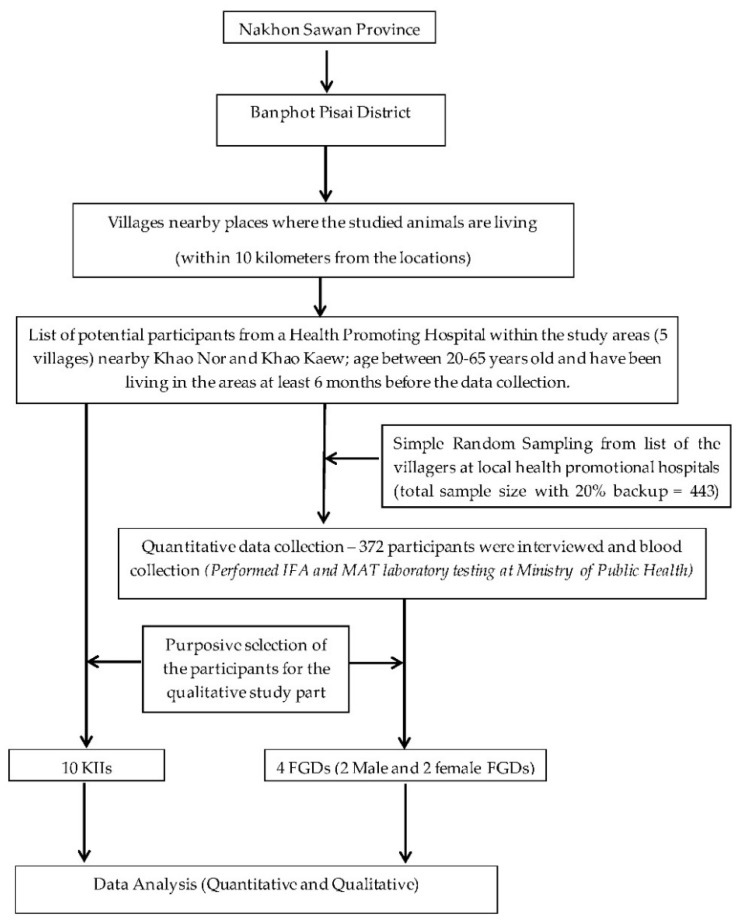
Flow chart for the data collection procedures. (KII = Key Informant Interview, FGD = Focus Group Discussion).

The quantitative questionnaire and discussion guides for KII and FGD were designed by modifying the questionnaires of previous studies [30,34,35,36]. The tools were refined after pretest activities with villagers in the areas with a prevalence of rodents, bats, and non-human primates in a province having similar sociodemographic and cultural contexts as the study areas. The data collection procedures are presented in Figure 2.

### 2.6. Study Variables and Data Analysis

Two outcome variables (leptospirosis seropositive and rodent contact behavior), as well as twelve independent variables, were studied. Descriptive statistics were then conducted on the overall samples, such as mean ± SD, median, and interquartile range (IQR) for continuous variables, and then numbers and percentages were used for categorical variables. Subsequently, the chi-square or Fisher’s exact tests were used to compare 2 categorical variables. Furthermore, univariate and multivariate logistic regression analyses were used to identify the outcomes resulting from the associated factors. Confidence intervals (CI) of 95% were also calculated for all indicators. The data were analyzed using the PASW Statistics for Windows, Version 18.0 (SPSS Inc., Chicago, IL, USA), after which a *p*-value < 0.05 was considered statistically significant.

All notes, feedback, and data for the KIIs and FGDs were entered onto a laptop computer. Audio recordings were also transcribed into Thai transcriptions. Then, qualitative analysis steps were performed using the following methods: (a) manually reviewing transcriptions using line-by-line analysis; (b) underlining the keywords based on the study objectives and themes of the discussion guides; (c) naming and grouping the keywords and contents; and (d) linking the reviews, interpreting the results, and providing recommendations using the triangulation approach [37].

### 2.7. Serological Tests

Subsequently, single serum samples were collected from participants and analyzed for anti-leptospiral antibodies using serological assays, including indirect immunofluorescence assay (IFA) and the gold-standard microscopic agglutination test (MAT). Positive IFA samples were randomly chosen to test agglutinating antibody titers against 24 serovars of live leptospires’ antigens [38]. The IFA and MAT assay protocols were conducted according to the Department of Medical Sciences, Ministry of Public Health, Thailand.

The IFA assay was performed using a mixture of local predominant *Leptospira* spp. cultures (serovars Bratislava, Autumnalis, Icterohaemorrhagiae, and Sejroe) as antigens. The leptospiral antigen was coated onto a 21-well slide glass and air-dried. The antigen was then fixed with cold acetone for 10 min, dried slide, and kept at −20 °C until used. The sera were twofold diluted from 1:50 to 1:3200 with PBS. Each diluted sample was applied on a slide and incubated at 37 °C in a moist chamber for 40 min. After washing the slide with PBS for 5 min, and doing this 3 times, secondary conjugated antibodies, i.e., rabbit anti-human IgM- and IgG conjugated FITC (Dako, Glostrup, Denmark) at a dilution of 1:40, were separately applied onto a slide. The reaction was allowed to take place at 37 °C, as previously described. The slide was observed under fluorescent microscopy. The serum samples (1:50 dilution) were initially screened for IgM and IgG antibodies to pool leptospiral antigens using the IFA assay. Positive serum samples were then diluted further to 2-fold serial dilutions to determine antibody titers. Following that, IgM- and IgG-positive serum samples were defined using antibody titers of ≥1:100.

The MAT assay was performed by using 10^8^ leptospires/mL of 24 serovars of *Leptospira* spp. cultures as an antigen. Briefly, 50 µL of each leptospiral serovar was applied into a micro titer plate containing 50 µL of PBS and 50 µL of diluted serum sample to make a final concentration of 1:50. The reaction was performed with a 2-fold serial diluted serum sample in which the final dilution was from 1:50 to 1:12, 800. The plate was gently mixed and incubated at room temperature for 120 min. The agglutination was observed under a dark-field microscope. The cut-off titers for the MAT-positive samples represented a 2 + agglutination titer (≥1:100), whereas the negative results showed titers <1:100.

## 3. Quantitative Survey Results

### 3.1. Profiles of Quantitative Survey Participants

Three hundred and seventy-two participants were randomly selected, who were Thais, and agreed to take part in the quantitative survey. Of these participants, 149 (40.1%) were male, and 223 (59.9%) were female. Their ages ranged between 20 and 65 years, with a mean age of 49.45 ± 12.07 years. We categorized the ages into two groups. Group 1 comprised ages between 20 and 45 years, which was considered the active working-age group, whereas Group 2, comprising participants aged over 45 years old, was considered the less active working-age group. Results showed that more than half of the participants (259, 69.6%) were older than 45 years, including 102 males and 157 females. However, 113 participants (30.4%), including 47 males and 66 females, were aged between 20 and 45 years (Table 1).

Data collected also revealed that most participants (224, 60.2%) completed their primary educational level, which was followed by those that completed their secondary education (62, 16.7%), vocational education (57, 15.3%), bachelor’s degree (20, 5.4%), and more than bachelor’s degree (1, 0.3%). Only one female participant in this study had completed more than a bachelor’s degree. Additionally, the educational attainment levels were categorized into two groups: a) those with basic educational attainment levels and b) those with higher than the standard educational attainment level. Most of the participants had basic educational attainment levels (232, 62.4%), including 92 males and 140 females, whereas 140 participants (57 males and 83 females) completed their education higher than secondary school level. They mainly were married (257, 69.1%), followed by a single (86, 23.1%), including those with other marital statuses such as divorced and widows (29, 7.8%). Furthermore, about half of the participants (188, 50.5%) reported their main occupation as being agriculture-related (i.e., farm or rice field farmers), followed by temporary employees, e.g., construction workers (62, 16.7%), vendors (41, 11.0%), having no occupation (33, 8.9%), office workers (14, 3.8%), other occupations (14, 3.8%), government officers (10, 2.7%), housemaids (8, 2.2%), and students (2, 0.5%). The main occupations were also grouped into agriculture- and non-agriculture-related occupations, of which about half of the participants (188, 50.5%) had main occupations involving agricultural tasks. In addition, 115 participants (77.2%) had their 3–6 persons in families per household, followed by ≤2 persons per household (25, 16.8%), and >6 persons per household (9, 6.0%). Most of them also had a low monthly family income, which was less than THB 15,000 or ≤USD 450 per month (111, 74.5%), followed by those with THB 15,001–40,000 or USD 450.10–1333.30 per month (35, 23.5%), and THB 40,000–70,000 or USD 1333.40–2333.30 per month (3, 2.0%). Moreover, 81 persons (54.4%) reported owning at least a car or truck in their households (Table 1).

### 3.2. Animal Contact Experiences

Two hundred and eighty-two participants (75.8%) reported at least one type of direct or indirect physical contact activity with rodents. The results also revealed that 90 (24.2%) of the participants had direct physical contact with one type of rodent through hunting (35, 9.4%), killing (41, 11.0%), preparing food (33, 8.9%), consuming cooked meats (12, 3.2%), feeding food (4, 1.1%), cleaning feces (17, 4.6%), or cleaning carcasses (33, 8.9%) (Table 2). It is important to note that those feeding food to rodents were individuals who farmed bandicoots to sell inside and nearby communities. In addition, 282 participants (75.8%) reported indirect physical contact with either one of the rodent types by seeing them without physical contact at any locations in their lifetime (114, 30.6%) and seeing them without any physical contact in households and communities in their lifetime (214, 57.5%) (Table 2).

### 3.3. Hunted, Killed, and Prepared Rodents as Food in the Past 12 Months

Table 3 shows the participants who killed rodents the most (41, 11.0%), followed by those that hunted (35, 9.4%) and prepared rodents as food (33, 8.9%). Among all reported rodent types, field rats were the most hunted (32, 8.6%), followed by killed (37, 9.9%) and prepared rodents as food (32, 8.6%).

### 3.4. Consumed Animals (Either Raw or Cooked) in the Past 12 Months

As per the prompted questions, 12 of the participants (3.2%) reported consuming the cooked meat of rodents. Brown rats were the most reported of the consumed cooked meat (6, 1.6%), followed by bandicoots (1, 1.1%) and squirrels (2, 0.5%) (Table 3). When probed with further questions, the participants recalled and reported different consumption activities, among which 198 of 372 participants reported rodent consumption (198, 53.2%). Most of them also reported consuming the rodent more than a decade ago (75, 37.9%), followed by 1.1–10 years (45, 22.7%), within 12 months (33, 16.7%), and the last month (31, 15.7%). Most participants reported that vendors killed these rodents (71, 35.6%). While the participants also killed rodents (50, 25.3%) by themselves, followed by neighbors (27, 13.6%), guardians (14, 7.1%), spouses (9, 4.5%), colleagues (9, 4.5%), and other persons, unspecified (9, 4.5%), including kids or children aged less than 18 years old (5, 2.5%), and hunters (4, 2.0%). Most of the participants also reported cooking rodents (98, 49.6%) by themselves, followed by neighbors (24, 12.1%), spouses (22, 11.1%), guardians (19, 9.6%), vendors (18, 9.1%), other persons, unspecified (9, 4.5%), and colleagues (4, 1.2.1%), including kids or children aged less than 18 years old (3, 1.5%), and hunters (1, 0.5%).

Furthermore, most participants consumed the rodents annually (61, 30.8%), followed by those who consumed these rodents every 3 months (46, 23.2%), every 6 months (26, 13.1%), and every month (22, 11.2%). Some participants also consumed rodents more than twice per year (17, 8.6%), including every week (4, 2.0%) and every day (1, 0.5%). Nevertheless, some consumed rodents as recently as a month ago (62, 31.3%), followed by within 12 months (55, 27.8%), within 1.1–10 years (43, 21.7%), more than 10 years (17, 8.6%), within the week (11, 5.6%), and the previous week (10, 5.1%).

Additionally, results showed that the most accessible rodent meat sources were from trapping in rice fields (86, 43.5%), followed by getting from vendors at the local markets (72, 36.4%), then from neighbors (21, 10.6%), forests (5, 2.5%), relatives (5, 2.5%), other sources, unspecified (4, 2.0%), within households (3, 1.5%), and no answers (2, 1.0%).

### 3.5. Being Bitten by Rodents, including the Period, and Practices toward Wounds

The participants reported that rodents did bite 25 persons (6.7%). They also reported being bitten by rodents themselves (13, 52.0%) during 1.1–10 years, followed by those bitten more than 10 years ago (8, 32.0%), and within the past 12 months (2, 8.0%). For treatment, they reported cleaning the wound with water immediately once they were bitten by the rodents (7, 28.0%), followed by meeting with doctors or nurses at health facilities (5, 20.0%), and cleaning the wound with soap, water, and antiseptic solutions immediately (4, 16.0%). Surprisingly, 5 of them (20%) did nothing when the rodents bit them.

### 3.6. Feeling toward Animals and Zoonotic Diseases

Among the 282 participants who reported at least one of direct and indirect physical contact activity with rodents, they reported varying feelings toward the feces of rodents in the following ways: some felt neutral (137, 48.6%), others felt dirty (40, 14.2%) or abominate (33, 11.7%), whereas others thought of the possibility of the feces causing infections (22, 7.8%), some were afraid of it (11, 3.9%), and the rest had other feelings (1, 0.4%).

They also reported feelings toward the carcasses of the rodents in the following ways: some felt neutral (115, 40.8%), others felt abominate (44, 15.6%) or dirty (38, 13.5%), whereas some thought of the possibility of carcasses causing infection (22, 7.8%), others were afraid of it (11, 3.9%), and the rest had other feelings (3, 1.0%).

### 3.7. Medical Histories

For the past 12 months, 372 participants reported the following self-identification symptoms: headache (197, 53.0%), high fever (132, 35.5%), muscle pain (119, 32.0%), nausea (53, 14.2%), vomiting (47, 12.6%), myalgia (42, 11.3%), and rash (36, 9.7%). In the past month, they also reported headaches (107, 28.8%), followed by muscle pain (73, 19.6%), feeling of high fever (39, 10.5%), myalgia (23, 6.2%), rash (16, 4.3%), nausea (11, 3.0%), and vomiting (7, 1.9%).

### 3.8. Leptospirosis Prevalence Study among the Study Subjects

Single serum samples (*n* = 372) were then examined for IgM and IgG antibody titers to predominantly local leptospiral serovars using the IFA assay. Of the 372 serum samples tested, 10 samples (2.7%) had positive IgM titers of ≥1:100 dilutions, and 44 samples (11.8%) gave antibody IgG titers of ≥1:100 (five persons were seropositive for both IgM and IgG). Details of the positive serum samples to *Leptospira* spp. by IFA assay is shown in Table 4.

Furthermore, IgM-positive samples (10 samples) were picked up to determine MAT titers. All samples had MAT titers of <1:100. In this study, out of the 372 participants, 169 had detectable IgM or IgG to leptospirosis (titers ≥ 1:50) with a seroprevalence of 45.5%. The IgM antibodies resulted in positives against selected predominant local serovar antigens, including Autumnalis, Bratislava, Icterohaemorrhagiae, and Sejroe.

Therefore, only 10 IgM results were used for further analysis steps since this study would like to determine the current leptospirosis infection due to current rodent and other animal exposure activities.

Nine sociodemographic variables, including seven knowledge, attitude, and practice (KAP) variables, in addition to twenty-one participants exposed to animals, were analyzed in the univariate analysis step against positive leptospirosis IgM titer laboratory results (Table 5). Among the 10 leptospirosis-positive IgM titer cases tested, 5 of them were males (50.0%). Their mean age was 47.7 years. Unfortunately, following the univariate analysis results, sociodemographic and KAP variables were insufficient for use in the next logistic regression step to determine the association between the risk factors and laboratory results (Table 5).

In addition, univariate analysis results in Table 5 showed that the participants reported contacting cats in the past 12 months (OR = 7.13, 95% CI 0.89–56.86, *p* = 0.05), hunting rodents of at least one type (OR = 4.42, 95% CI 1.09–17.93, *p* = 0.06), hunting field rats (OR = 4.92, 95% CI 1.21–20.05, *p* = 0.05), killing rodents of at least one type (OR = 3.65, 95% CI 0.91–14.72, *p* = 0.09), and feeding food to bandicoots (OR = 41.22, 95% CI 21.62–78.60, *p* = 0.03) (Table 6). Unfortunately, this behavior was not statistically significant.

### 3.9. Rodent Exposure Levels

For the rodent exposure-level analysis, two variables (gender and monthly household income) were eligible for the logistic regression analysis (Table 7). The logistic regression results showed that the male gender was statistically significant with reports of encountering rodents about 3.14 times (Adjusted OR = 3.14, 95% CI 1.91–5.14, *p* < 0.001) (Table 7). Additionally, villagers who had low monthly household incomes (≤THB 15,000 or USD 450) had lowered encounters with rodents, when compared with those who had monthly income levels about 0.57 times higher (Adjusted OR = 0.57, 95% CI 0.33–0.99, *p* = 0.04) (Table 8).

## 4. Qualitative Data Collection Results

### 4.1. Profiles of Qualitative Study Participants

A total of 35 persons participated in the study, including 11 persons (6 males and 5 females) who participated in KIIs, whereas 24 participants (12 males and 12 females) participated in the FGDs. The participants’ mean age ± SD was 45.0 ± 9.26 years, with age ranges (minimum and maximum) between 21 to 58 years old. Most of them were agriculture-related farmers (37.1%), followed by food vendors (25.7%), government employees (17.1%), self-employed and seasonal workers (14.3%), and monks (5.7%). However, among the 11 KII participants, 2 non-human primate-feeding food vendors, 2 bat guano (feces) collectors, 2 rodent hunters, 2 community leaders, and 3 government staff from the municipality, conservation office, and health-promoting hospital were participants. The participants described their contact characteristics with rodents, bats, and non-human primates differently.

### 4.2. Rodent Contact Characteristics among the Qualitative Participants

Two rodent hunters aged between 45 and 55 years old were interviewed. Their main occupations were farming, whereas the other was a temporary employee who assisted in farming activities around the subdistrict.

These participants reported contact with different types of rodents, especially great bandicoots, field rats, roof rats, Ryukyu mice, Polynesian rats, brown rats, also known as rats or Noo, squirrels, and tree shrews. They found and directly contacted field rats, bandicoots, and Ryukyu mice around their rice fields and farms at least once a month while working. However, regularly, Ryukyu mice and brown rats were found inside and around their households.

From their reports, great bandicoots were also among the rodents that the villagers found in their areas. These were the heaviest rodents found, which weighed around 1 kg. Furthermore, they were characterized by stiff hair and stayed around rice fields. Additionally, their diet includes snails, cassava, and rice, and they can dive underwater.

Reports also revealed that field rats, or bandicoots, had a medium size of around 600 g and liked to stay together with their family members. They also liked to stay in rice fields and destroy crops, including rice. “Bandicoot likes to stay with their family members”, (a male FGD participant, FGD002).

However, the participants concluded that great bandicoots, field rats, and small bandicoots were similar species. “Field rats, bandicoots, and great bandicoots are the same ones”, (a male FGD participant, FGD001).

Roof rats were the smallest rodents reported, with these rodents having three giant fingers and living in rice fields. They were also reported to have white hair under their abdomens and have long sharp mouths. According to the reports, they lived on trees and ate everything, including fruits, corn, cassava, and snails. Rodent hunters do not like to have this type of rodent because they are tiny.

Alternatively, reports revealed that Polynesian rats and Ryukyu mice were small and liked finding their food at nighttime, making people call them Ghost Rats or Soricines (Shrews). Polynesian rats also make a sound that sounds like “Jeed Jeed”. However, these rats can run the fastest.

### 4.3. Rodent Contact Activities and Their Perception toward Rodents

Various rodent contact activities were reported among the villagers, such as hunting, consumption, and selling them to their friends and neighbors. Males, who like to drink alcohol, were prominent persons who hunted, killed, and prepared the field rodents before cooking or selling them to their friends or neighbors. Meats were also sold fresh (after removing the intestine and internal organs) or cooked, such as in a curry or fry. However, hunters touched, hunted, killed, and prepared the rodents with bare hands and cleaned their hands with only fresh water without any soap, because some of them (hunters) do not think these rodents have diseases. Nevertheless, they perceived that leptospirosis infections occurred because of the uncooked rodent meat.

Reports also revealed that the rodent hunting season is from October to February. Hunters have therefore sold them around THB 150 or USD 30 per kilogram. Menus for cooking the rodents are dried frying with spicy ingredients, frying with vegetables, mixing their meats with curry pastes, and frying with garlic or pepper. Thus, every gender and age group has consumed rodent meat together, and if any family does not eat the rodents, those family members will not consume the rodents either.

Nowadays, people have less contact with rodents than in previous times because many rodent hunters exist and there are new methods for plowing rice fields using a tractor that destroys many rodent babies in the fields. Furthermore, many snakes that eat rodents also live in the fields.

### 4.4. Benefits and Disadvantages of Rodents

Benefits of rodents were also reported, such as their serving as a source of additional income by selling these rodents and their meat as food. In contrast, rodents also destroy their belongings, crops, and plants. Some participants also reported that rodents transmitted leptospirosis infections.

### 4.5. Mitigation Strategies to Be Protected from Rodents and Diseases

**Figure 3:**
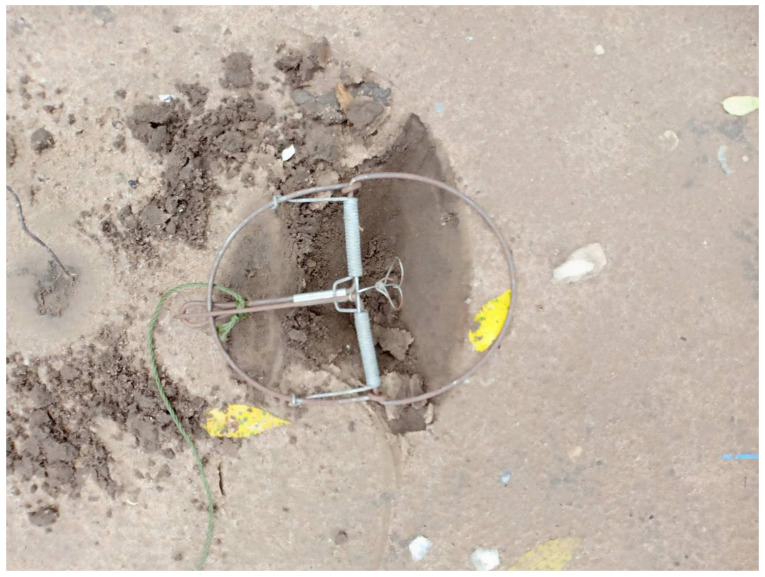
Trap using for catching rodents obtained from the northeastern region of Thailand.

Feedback from the participants also revealed that they used several methods to protect rodents from destroying their belongings and crops. In households, they used traps, glue traps, and ratsbane to eliminate the rats. In contrast, they used the tractor to kill them during the harvesting period, killing more than 10 kg at a time. The villagers also reported using boots while they worked on the fields and traps that they got from the Chaiyapom province (Figure 3).

Additionally, the local health-promoting hospital (HPH) previously organized a campaign to promote information on leptospirosis to inform the villagers on the use of appropriate prevention while they worked in their fields and had wounds. This prevention comprised wearing long boots, protecting themselves from wounds, and if they had any fever or relevant symptoms, they were mandated to seek treatment at the HPH immediately.

## 5. Discussion and Conclusions

This study showed that participants came into contact with rodents of different species based on their locations, occupations, and activities. Furthermore, the rodents were hunted, killed, prepared as food, and consumed cooked in several menus. Male hunters were most exposed to the rodents because they hunted, killed, cooked, and sold the hunted rodents to their friends and neighbors.

This cross-sectional survey also showed that only the male gender showed significant (OR = 3.137, 95% CI 1.914–5.139, *p* < 0.001) statistical rates of encountering rodents. This result was in line with results of a study was conducted at the Mpumalanga province of South Africa, which found that the male gender-typed tasks included those associated with livestock and poultry husbandry, hunting, and slaughtering wildlife and rodent control [39]. Another study at the Khon Kaen Province, a northeastern province of Thailand, in 2011 also revealed that males were associated with highly reported rates of rodent consumption [31].

This current study also found that persons with high monthly household incomes (more than THB 15,000 or around USD 450) encountered rodents about twice as frequently as persons with lower income levels. The findings were comparable with the Khon Kaen study [34] that having a car and living in a home with wooden walls were positively and marginally significantly associated with rodent contact in or near the home. This Khon Kaen study indicated that possessing a car and living in a house with wooden walls might imply a higher socioeconomic status.

### 5.1. Rodent as Food

This study also showed that the rodent consumption rate for the past 12 months through prompted questions was 3.2%, while through the probed question, the result was 53.2%. These results are compatible with previous studies in various locations, such as in Cambodia, Laos, Myanmar, parts of the Philippines and Indonesia, other provinces of Thailand, Ghana, China and Vietnam where people consumed rodents as food [31,32,40,41].

The Khon Kaen study [31] also showed that 113 (56.2%) of 201 participants (39 females and 74 males) consumed at least one type of rodent. Among these 113 participants, 104 (92.0%), 4 (3.5%), 6 (5.3%), 33 (29.2%), 19 (16.8%), and 3 (2.7%) consumed only rats (no specific species), including field rats, squirrels, burrowing squirrels, flying squirrels, and porcupines, respectively. Another study [32] in 29 villages of the Khamkeuth District in the Bolikhamxay Province of Laos PDR in 2013 found that among the 584 study participants, 39.9% of them hunted or captured rodents and it also showed that 77.7% of them prepared rodents as food, whereas 86.3% consumed rodents as food. The above could be an evidence-based information that the rodent consumption behavior of the northern region of Thailand and Lao PDR people was similar.

A study in the Republic of Guinea [40] showed that peridomestic rodents were hunted as a protein source by 91.5% of the Gueckedou Prefecture population. Additionally, a study in Benin [42] showed that more than 75.0% of the village populations ate grasscutters, giant rats, grass rats, and crested porcupines. In comparison, other rodent types consumed by 51–75% of the Beninese village population were rodents, as these rodents were considered animal protein sources.

However, this present study had a lower prevalence of rodent consumption than the previous mentioned studies, especially with the prompted question method. The result was different because the study participants were afraid to inform the researchers of their actual consumption behavior at first. Additionally, the cultures of the people living in the central region were different from those living in the Isan (northeastern) region of Thailand or Lao PDR, which had more exposure to and consumption of wildlife animals than the people living in the current study areas. However, we consider that this is the first study that reported rodent consumption rates among villagers living in the central region of Thailand.

### 5.2. KAP and Mitigation Measures for Interacting with Rodents

This study found fascinating information that hunters touched, hunted, killed, and prepared the rodents as food with bare hands and cleaned their hands with only fresh water without any soap. According to our findings, some of them (hunters) did not believe rodents carried diseases. They also believed that leptospirosis infections occurred because of consuming the uncooked rodent meat. Therefore, even though the HPH previously provided leptospirosis health promotion campaigns, the knowledge might not have reached these hunters, which could be barriers to them changing their KAP. The findings were consistent with those of an Indian study [43], which also showed that populations in both rural and urban areas were less knowledgeable about risk factors and practiced poor rodent contact behaviors. Interestingly, points from this India study showed that although education had a significant impact on the knowledge and attitudes of the urban population, their practices did not improve with education.

### 5.3. Seroprevalence of Leptospirosis among the Study Participants

As several scoping visits and reviews of health statistics at HPHs and the PHO reported villagers had close-contact activities with rodents. The health data also showed some cases of fever of unknown origin (FUO) in the study areas. Additionally, the villagers had agriculture-related occupations that were possible risk factor for leptospirosis infections. Therefore, this study used leptospirosis as a “proxy indicator” to determine the exposure activities of villagers to animals in the study areas to obtain the seroprevalence rate among people who have close-contact activities with the rodents.

A previous study showed that laboratory criteria for seroprevalence studies among subjects varied, ranging from a MAT titer of ≥1:20–1:200, with an average MAT titer of ≥1:80. ELISA or IFA were used to detect the presence of specific IgM antibodies in this study [44]. In our current study, individuals who presented specific IgM and IgG antibody titers of ≥1:100 were considered seropositive samples and samples that had an IFA titer of ≥1:100 (*n* = 39) on either IgM or IgG antibodies were considered seropositive samples. As a result, the prevalence of leptospirosis among the study subjects at the Banphot Pisai district was 10.5% (39 of 372 study participants). If the cut-off titer for an IgG antibody was set at ≥1:400 in accordance with leptospirosis diagnostic criteria, seroprevalence could be calculated to be 2.7% among only IgM-positive samples.

Additionally, the study was done in an area that is non-endemic to leptospirosis during the rainy season (July to September) in 2019, which might flood in the study district’s wet season. As a result, villagers were expected to have been exposed to *Leptospira* pathogens from infected animals, contaminated environments, or occupation-related activities. IgG seroprevalence of leptospirosis among participants was also proposed from the pre-existing IgG antibody in response to previous pathogen exposure, whereas IgM seropositive samples among subjects would also arise from asymptomatic infection. Similarly, additional studies showed a seroprevalence of leptospirosis in human ranging from 0.0% to 83.5%, depending on disease endemicity and risks of exposure to animal reservoirs [44]. Thus, Chadsuthi et al. [45] found a 23.7% MAT-positive seroprevalence among suspected leptospirosis patients in Thailand during 2010–2015, with the predominant serovars Shermani, Bratislava, Panama, and Sejroe, whereas leptospirosis detected a 28.0% IgG seroprevalence in young Thai men during 2007–2008 [46]. The identification of the pathogenic serovar has larger implications for the epidemiological investigation of animal reservoirs and potential zoonotic transmission. In this current investigation, IgM antibodies were positive against selected predominant local serovar antigens, including Autumnalis, Bratislava, Icterohaemorrhagiae, and Sejroe. Furthermore, infecting serovars in domestic animals in Thailand were Bratislava, Mini, and Sejroe. In a similar study, a survey of cattle and buffaloes in the lower northeastern areas revealed the dominant infecting serovars as Shermani, Ranarum, and Tarassovii [47], whereas a survey in stray dogs showed an 84.0% seroprevalence to serovar Batavia. Hence, rodents are the main source of infected serovars (Pyrogenes, Batavia, Autumnalius, Javanica, and Australis) [48].

The seroprevalence rate for IgM and IgG-positive samples in this study was relatively high (45.4%), while the seroprevalence rate for positive IgM was low among study participants (2.4%). These values were less comparable to those found in previous investigations in Thailand, especially in northeastern Thailand [49,50]. Similarly, the study’s reduced seroprevalence rate might be attributed to the villagers’ receiving health education from HPH officials several years ago, particularly during Thailand’s leptospirosis epidemics. As a result, the villagers have been employing a variety of human protection measures (e.g., boots) and mitigation strategies (e.g., traps and trac-tors) to eradicate rats from their rice and agriculture fields. Moreover, unlike hospital-based studies, this study was undertaken among the general community population. Therefore, the numbers should be even lower than those reported in other research, particularly in areas with endemic leptospirosis.

A previous study [51] revealed that the national leptospirosis incident rates declined from 23.1 to 5.9 per 100,000 people. We assumed that our study results were the outcome of the national and local health offices’ active and repeated communication and health promotion intervention activities to promote systematic rodent control, educate villagers as to proper sanitation and hygiene practices, and promote the use of boots and gloves when working on farms. Therefore, the seroprevalence rate of 2.7% was understandable, particularly in locations with no past leptospirosis outbreaks. In addition, this result was the first report of leptospirosis seroprevalence in this location, which the data can be used to guide future relevant research in this province and the central region.

Additionally, this study demonstrated by univariate analysis that study participants came into contact with a variety of rodents associated with leptospirosis infections. They did, however, interact with other animal species, including domestic (e.g., cats) and wild (e.g., bats and long-tailed macaques). This finding is thought to have caused leptospirosis infections, as these animals are considered leptospirosis reservoirs and were in line with previous studies indicating that close contact with domestic and wild animals increased the chance of leptospirosis infections [28]. Therefore, a follow-up study with a larger sample size and individuals who have had interaction with various animal species should be investigated to confirm the aforementioned results.

### 5.4. Study Limitations

This study asked about their previous experiences contacting rodents; therefore, recall bias would be one of the limitations. The participants might not recall their experiences, locations, and contact times with the rodents.

This current study found a low seroprevalence rate, but it was within expectations since the current study areas had no previous reports of leptospirosis outbreaks, and the target populations were not considered leptospirosis patients. In addition, even though the villagers were exposed to rodents regularly, especially in their agricultural settings, the qualitative study results showed that the villagers were provided health education on leptospirosis through local health staff several years ago during Thailand’s leptospirosis outbreaks and flooding events in this area which the villagers consequently have been using various personnel protective methods (e.g., booths) and mitigation strategies (e.g., traps and tractors) to eliminate the rats in their rice and farm fields. Therefore, the leptospirosis seroprevalence rate could be lower than those reported in other studies, especially in the areas where leptospirosis is endemic.

Moreover, the results of this study could not represent Nakhon Sawan province but were only for Khao Nor and Khao Kaew, which have various types of domestic and wildlife (e.g., bats and non-human primates) animals in the areas. However, these findings may be generalized to locations with the same types of animals or similar environmental and animal habitat characteristics, such as Lopburi province.

### 5.5. Recommendations

We recommend that communication intervention should be planned to provide appropriate knowledge and promote an appropriate attitude toward animals, contact characteristics, and zoonotic diseases, including how to practice these protocols appropriately, especially among persons who have had close contact with rodents, such as hunters and vendors selling rodent meat.

Health education activities about zoonotic diseases and mitigation measures led by local health facilities should also be implemented. Furthermore, local health staff, authorities, and village health volunteers (VHVs) should be educated on the possible zoonotic diseases that can be contracted from animals, in addition to providing training on mitigation measures and motivating them to communicate with villagers about the impact of contracting any diseases from animals, especially rodents and other types of domestic and wildlife animals. Various mass and social media communication platforms should also be considered as a medium to implement these behavioral change intervention activities.

The results should inform relevant policymakers to educate the public on having appropriate practices for direct and indirect physical contact with rodents and other animals. These may assist in reducing the risk of leptospirosis and other rodent-borne diseases in the study area and Thailand.

Additionally, further research on seroprevalence studies conducted among populations who have had contact with various animals (both domestic and wildlife) should be conducted. Based on the results of this current study, people who had contact with cats, hunted any species of rodents or field rats, those that killed rodents, and those that cleaned the feces of bandicoots had a potential risk of leptospirosis infections per univariate analysis results because these animals are considered hosts for leptospirosis. A larger sample size should be considered for future studies. The benefits of the seroprevalence rates should also be considered for further studies concerning environmental factors and animals.

## Figures and Tables

**Table 1:** Sociodemographic profile distribution by gender (*n* = 372).

SES Variables	Males (149, 40.1%) *n* (%)	Females (223, 59.9%) *n* (%)	Total (372, 100.0%) *n* (%)
**Age groups**			
20–30 years old	19 (12.8%)	26 (11.7%)	45 (12.1%)
31–40 years old	14 (9.4%)	21 (9.4%)	35 (9.4%)
41–50 years old	31 (20.8%)	46 (20.6%)	95 (20.7%)
51–60 years old	59 (39.6%)	87 (39.0%)	92 (39.2%)
61–65 years old	26 (17.4%)	43 (19.3%)	69 (18.5%)
**Age groups (Cut-off age at 45 years old)**			
20 to 45 years old	47 (31.5%)	66 (29.6%)	113 (30.4%)
>45 years old	102 (68.5%)	157 (70.4%)	259 (69.9%)
Range	20–65 year old	20–65 year old	20–65 year old
Mean + SD	49.34 ± 12.15	49.52 ± 12.06	49.45 ± 12.07
**Marital status**			
Single	34 (22.8%)	52 (23.3%)	86 (23.1%)
Married	107 (71.1%)	150 (67.3%)	257 (69.1%)
Other (divorced or widows)	8 (2.2%)	21 (9.4%)	29 (7.8%)
**Educational attainment levels**			
No formal education	2 (1.3%)	6 (2.7%)	8 (2.2%)
Primary school	90 (60.4%)	134 (60.1%)	224 (60.2%)
Secondary school	34 (22.8%)	28 (12.6%)	62 (16.7%)
Vocational education	18 (12.1%)	39 (17.5%)	57 (15.3%)
Bachelor’s degree	5 (3.4%)	15 (6.7%)	20 (5.4%)
More than Bachelor’s degree	0 (0.0%)	1 (0.4%)	1 (0.3%)
**Main occupations**			
No occupation	9 (6.0%)	24 (10.8%)	33 (8.9%)
Agriculture	79 (53.0%)	109 (48.9%)	188 (50.5%)
Temporary employee	35 (23.5%)	27 (12.1%)	62 (16.7%)
Office worker	5 (3.4%)	9 (4.0%)	14 (3.8%)
Vendor	11 (7.4%)	30 (13.5%)	41 (11.0%)
Government officer	1 (0.7%)	9 (4.0%)	10 (2.7%)
Housemaid	0 (0.0%)	8 (3.6%)	8 (2.2%)
Student	0 (0.0%)	2 (0.9%)	2 (0.5%)
Other occupation	9 (6.0%)	5 (2.2%)	14 (3.8%)
**Number of family members**			
≤2 persons	25 (16.8%)	51 (22.9%)	76 (20.4%)
3–6 persons	115 (77.2%)	159 (71.3%)	274 (73.7%)
>6 persons	9 (6.0%)	13 (5.8%)	22 (5.9%)
**Monthly household income**			
≤THB 15,000 or ≤USD 450	111 (74.5%)	172 (77.1%)	283 (76.1%)
THB 15,001–40,000 or USD 450.10–1333.30	35 (23.5%)	46 (20.6%)	81 (21.8%)
THB 40,001–70,000 or USD 1333.40–2333.30	3 (2.0%)	5 (2.2%)	8 (2.2%)
**Owned a car or a truck**			
Yes	81 (54.4%)	135 (60.5%)	216 (58.1%)
No	68 (45.6%)	88 (39.5%)	156 (41.9%)

**Table 2:** Frequency of rodent contact activities (*n* = 372).

Contact Activities	Rodent Contact Activity Rates *n* (%)
**Direct or indirect physical contact activities with either one of the rodent types**	***282 (75.8%)***
***Direct physical contact with either one of the rodent types***	***90 (24.2%)***
Hunted	35 (9.4%)
Killed	41 (11.0%)
Prepared rodents as food	33 (8.9%)
Consumed cooked meat	12 (3.2%)
Fed food to rodents	4 (1.1%)
Cleaned feces	17 (4.6%)
Cleaned carcasses	33 (8.9%)
***Indirect physical contact with either one of the rodent types***	***282 (75.8%)***
Had seen rodents without physical contact at any locations in their lifetime	114 (30.6%)
Had seen rodents without physical contact in households and communities in their lifetime	214 (57.5%)

**Table 3:** A summary of the hunted, killed, and prepared rodents as food, consumed raw, and cooked for the past 12 months (*n* = 372).

Rodent Types (Scientific Names)	Hunted	Killed	Prepared as Food	Consumed Cooked Meat
	(*n*, %)	(*n*, %)	(*n*, %)	(*n*, %)
At least one species of rodents	35 (9.4%)	41 (11.0%)	33 (8.9%)	12 (3.2%)
Field rat (Rattus argentiventer)	32 (8.6%)	37 (9.9%)	32 (8.6%)	0 (0.0%)
Bandicoot (Peramelemorphia)	3 (0.8%)	4 (1.1%)	4 (1.1%)	4 (1.1%)
Brown rat (Rattus norvegicus)	1 (0.3%)	3 (0.8%)	0 (0.0%)	6 (1.6%)
Ryukyu mouse (Mus caroli)	1 (0.3%)	1 (0.3%)	0 (0.0%)	0 (0.0%)
Squirrels (Sciuridae)	2 (0.5%)	0 (0.0%)	0 (0.0%)	2 (0.5%)
Tree shrew (Scandentia)	1 (0.3%)	0 (0.0%)	0 (0.0%)	1 (0.3%)

**Table 4:** Positive serum samples to *Leptospira* spp. by IFA assay.

Serum ID	IgM Titers	IgG Titers
56	1:100	<1:50
171	1:100	<1:50
174	1:200	<1:50
196	1:100	1:100
197	1:100	1:100
198	1:100	1:50
199	1:100	1:200
200	1:100	1:50
203	1:100	1:100
273	1:100	1:200

**Table 5:** Univariate analysis of IgM for leptospirosis laboratory results concerning the study population’s sociodemographic characteristics, and Knowledge, Attitude, and Practice (KAP) variables (*n* = 372).

Variables	Total (*n* = 372)	Leptospirosis Results		Crude OR (95% CI)	*p*-Value
		Positive (*n* = 10)	Negative (*n* = 362)		
Sociodemographic (SES) information (9 variables)					
***Gender***					
**Male**	149 (40.1%)	5 (50.0%)	144 (39.8%)	1.51 (0.43–5.32)	0.53
**Female**	223 (59.9%)	5 (50.0%)	218 (60.2%)	1(ref)	
***Age groups***					
**20–45 years**	113 (30.4%)	3 (30.0%)	110 (30.4%)	0.98 (0.25–3.87)	1.00
**>45 years**	259 (69.6%)	7 (70.0%)	252 (69.6%)	1(ref)	
***Marital status***					
**Married**	257 (69.1%)	8 (80.0%)	249 (68.8%)	1.86 (0.38–8.69)	0.73
**Other marital status**	115 (30.9%)	2 (20.0%)	113 (31.2%)	1(ref)	
***Educational attainment***					
**Basic educational attainment level**	232 (62.4%)	5 (50.0%)	227 (62.7%)	0.60 (0.17–2.09)	0.51
**Other educational attainment levels**	140 (37.6%)	5 (50.0%)	135 (37.3%)	1(ref)	
***Main occupations***					
**Agriculture-related occupation**	188 (50.5%)	7 (70.0%)	181 (50.0%)	2.33 (0.59–9.17)	0.34
**Non-agriculture-related occupation**	184 (49.5%)	3 (30.0%)	181 (50.0%)	1(ref)	
***Secondary occupations***					
**Agriculture-related occupation**	39 (10.5%)	2 (20.0%)	37 (10.2%)	2.20 (0.45–10.73)	0.28
**Non-agriculture-related occupation**	333 (89.5%)	8 (80.0%)	325 (89.8%)	1(ref)	
***Numbers of family members***					
**≤2 persons**	76 (20.4%)	1 (10.0%)	75 (20.7%)	0.43 (0.05–3.41)	0.69
**>2 persons**	296 (79.6%)	9 (90.0%)	287 (73.2%)	1(ref)	
***Monthly household income***					
**≤THB 15,000 or ≤USD 450**	283 (76.1%)	9 (90.0%)	274 (75.7%)	2.89 (0.36–23.14)	0.46
**>THB 15,001 or >USD 450**	89 (23.9%)	1 (10.0%)	88 (24.3%)	1(ref)	
***Owned a car or a truck***					
**Yes**	216 (58.1%)	6 (60.0%)	210 (58.0%)	1.09 (0.30–3.91)	1.00
**No**	156 (41.9%)	4 (40.0%)	152 (42.0%)	1(ref)	
***Knowledge, Attitude, and Practice (KAP) (7 variables)***					
***Knowledge score on animals and zoonotic diseases***					
**≥80%**	228 (61.3%)	6 (60.0%)	222 (61.3%)	0.95 (0.26–3.41)	1.00
**<80%**	144 (38.7%)	4 (40.0%)	140 (38.7%)	1(ref)	
***Attitude score toward rodents***					
**≥80%**	66 (17.7%)	0 (0.0%)	66 (18.2%)	1.03 (1.01–1.06)	0.22
**<80%**	306 (82.3%)	10 (100.0%)	296 (81.8%)	1(ref)	
***Practice score toward rodents***					
**≥80%**	24 (6.5%)	0 (0.0%)	24 (6.6%)	1.03 (1.01–1.05)	1.00
**<80%**	348 (93.5%)	10 (100.0%)	338 (93.4%)	1(ref)	

OR = Odds Ratio, CI = Confidence Interval, ref = Reference values.

**Table 6:** Univariate analysis of leptospirosis laboratory results concerning animal contacts (*n* = 372).

Variables	Total	Leptospirosis Results		Crude OR (95% CI)	*p*-Value
	(*n* = 372)	Positive (*n* = 10)	Negative (*n* = 362)		
***Contacted with the animals in the past 12 months***					
***Cats***					
**Yes**	211 (56.7%)	9 (90.0%)	202 (55.8%)	7.13 (0.89–56.86)	0.05 *,**
**No**	161 (43.3%)	1 (10.0%)	160 (44.2%)	1(ref)	
***Hunted animals in the past 12 months***					
***At least one type of rodents***					
**Yes**	35 (9.4%)	3 (30.0%)	32 (8.8%)	4.42 (1.09–17.93)	0.06 *,**
**No**	337 (90.6%)	7 (70.0%)	330 (91.2%)	1(ref)	
***Field rat***					
**Yes**	32 (8.6%)	3 (30.0%)	29 (8.0%)	4.92 (1.21–20.05)	0.05 *,**
**No**	340 (91.4%)	7 (70.0%)	333 (92.0%)	1(ref)	
***Killed animals in the past 12 months***					
***At least one type of rodents***					
**Yes**	41 (11.0%)	3 (30.0%)	38 (10.5%)	3.65 (0.91–14.72)	0.09 *,**
**No**	331 (89.0%)	7 (70.0%)	324 (89.5%)	1(ref)	
***Fed food to rodents***					
***Bandicoot***					
**Yes**	1 (18.8%)	1 (10.0%)	0 (0.0%)	41.22 (21.62–78.60)	0.03 *,**
**No**	371 (81.2%)	9 (90.0%)	362 (100.0%)	1(ref)	

* *p*-value < 0.15 as cut-off point to further analysis of the logistic regression. ** *p*-value < 0.05 as statistically significant. OR = Odds Ratio, CI = Confidence Interval.

**Table 7:** Univariate analysis of rodent exposure levels concerning sociodemographic characteristics, and Knowledge, Attitude, and Practice (KAP) variables.

Variables	Total (*n* = 372)	Rodent Exposure Level		Crude OR (95% CI)	*p*-Value
		Direct (*n* = 90)	Indirect (*n* = 282)		
***Sociodemographic (SES) information (9 variables)***					
***Gender***					
**Male**	149 (40.1%)	55 (61.1%)	94 (33.3%)	3.14 (1.92–5.13)	<0.01 *,**
**Female**	223 (59.9%)	35 (38.9%)	188 (66.7%)	1(ref)	
***Age groups***					
**20–45 years**	113 (30.4%)	31 (34.4%)	82 (29.1%)	1.28 (0.77–2.12)	0.36
**>45 years**	259 (69.6%)	59 (65.6%)	200 (70.9%)	1(ref)	
***Marital status***					
**Married**	257 (69.1%)	63 (70.0%)	194 (68.8%)	1.06 (0.63–1.77)	0.90
**Other marital status**	115 (30.9%)	27 (30.0%)	88 (31.2%)	1(ref)	
***Educational attainment levels***					
**Basic educational attainment level**	232 (62.4%)	55 (61.1%)	177 (62.8%)	0.93 (0.57–1.16)	0.80
**Other educational attainment levels**	140 (37.6%)	35 (38.9%)	105 (37.2%)	1(ref)	
***Main occupations***					
**Agriculture-related occupation**	188 (50.5%)	51 (56.7%)	137 (48.6%)	1.38 (0.86–2.23)	0.19
**Non-agriculture-related occupation**	184 (49.5%)	39 (43.3%)	145 (51.4%)	1(ref)	
***Secondary occupations***					
**Agriculture-related occupation**	39 (10.5%)	10 (11.1%)	29 (10.3%)	1.09 (0.51–2.34)	0.84
**Non-agriculture-related occupation**	333 (89.5%)	80 (88.9%)	253 (89.7%)	1(ref)	
***Numbers of family members***					
**≤2 persons**	76 (20.4%)	20 (22.2%)	56 (19.9%)	1.15 (0.65–2.05)	0.65
**>2 persons**	296 (79.6%)	70 (77.8%)	226 (80.1%)	1(ref)	
***Monthly household income***					
**≤THB 15,000 or ≤USD 450**	283 (76.1%)	61 (67.8%)	222 (78.7%)	0.57 (0.34–0.96)	0.05 *,**
**>THB 15,001 or >USD 450**	89 (23.9%)	29 (32.2%)	60 (21.3%)	1(ref)	
***Sociodemographic (SES) information (9 variables)***					
***Owned a car or a truck***					
**Yes**	216 (58.1%)	53 (58.9%)	163 (57.8%)	1.05 (0.65–1.69)	0.90
**No**	156 (41.9%)	37 (41.1%)	119 (42.2%)	1(ref)	
***Knowledge, Attitude, and Practice (KAP) (7 variables)***					
***Knowledge score on animals and zoonotic diseases***					
**≥80%**	228 (61.3%)	57 (63.3%)	171 (60.6%)	1.12 (0.69–1.83)	0.71
**<80%**	144 (38.7%)	33 (36.7%)	111 (39.4%)	1(ref)	
***Attitude score toward rodents***					
**≥80%**	66 (17.7%)	17 (18.9%)	49 (17.4%)	1.11 (0.60–2.04)	0.75
**<80%**	306 (82.3%)	73 (81.1%)	233 (82.6%)	1(ref)	
***Practice score toward rodents***					
**≥80%**	24 (6.5%)	4 (4.4%)	20 (7.1%)	0.61 (0.20–1.83)	0.47
**<80%**	348 (93.5%)	86 (95.6%)	262 (92.9%)	1(ref)	

* *p*-value < 0.15 as a cut-off point to further analysis of the logistic regression. ** *p*-value < 0.05 as statistically significant. OR = Odds Ratio, CI = Confidence Interval.

**Table 8:** Comparison between results of the univariate and multivariate of rodent exposure levels.

Factors	Univariate		Multivariate	
	Crude OR (95% CI)	*p*-Value	Adjusted OR (95% CI)	*p*-Value
***Gender***				
Male	3.143 (1.924–5.134)	<0.001 **	3.137 (1.914–5.139)	<0.001 **
Female	1(ref)			
***Monthly household income***				
≤THB 15,000 or ≤USD 450	0.568 (0.336–0.962)	0.046 **	0.57 (0.332–0.985)	0.044 **
>THB 15,001 or >USD 450.10	1(ref)			

** Statistically significant at *p* < 0.05. OR = Odds Ratio, CI = Confidence Interval. THB = Thai Baht and UCD = United States dollar.

## Data Availability

The data presented in this study are available on request from the corresponding author.
